# Recurrence of post burn contractures of the elbow and shoulder joints: experience from a ugandan hospital

**DOI:** 10.1186/s12893-015-0089-y

**Published:** 2015-09-09

**Authors:** Deo Darius Balumuka, George William Galiwango, Rose Alenyo

**Affiliations:** Mbarara University of Science and Technology, PO Box 1410, Mbarara, Uganda; CoRSU hospital, PO Box 46, Kisubi, Uganda; College of Health Sciences School of Medicine, Makerere University, PO Box 7062, Kampala, Uganda

**Keywords:** Recurrence, Post Burn Contractures, Elbow joint, Shoulder joint, PBC

## Abstract

**Background:**

Recurrence of post-burn contractures, following inadequate management of post-burn contractures (PBC), is under reported. It is associated with multiple operations and an increased cost to patients and their families. The purpose of this study was to determine the frequency of recurrence of PBC of the shoulder and the elbow joint three months after surgical intervention and the associated risk- factors.

**Methods:**

This was a prospective cohort study conducted at CoRSU hospital from March 2012 to November 2014. All patients with PBC of the elbow and/or shoulder joint who consented to be in the study and met the inclusion criteria were enrolled. Data was collected using a pretested, coded questionnaire. A goniometer was used to measure the active range of motion of the involved joint. The measurements were recorded in degrees. The data was analysed with STATA version 12.1.

**Results:**

58 patients were enrolled consecutively in the study. There were 36 females and 22 males, with a female to male ratio of 1.6:1. The age range was 0.75–45 years, with a median age of 5 years. The average age at the time of injury was 3.4 years. The most common cause of initial burn injury was scalding. The average number of joints involved per patient was two. There was a high incidence of recurrence of PBC (52 %) among the participants. The shoulder had the highest frequency of recurrence at 67 %. The elbow joint had a frequency of recurrence of 27 %. All participants with both elbow and shoulder joint involvement had PBC recur. The risk factors for recurrence were flame burn (*p* = 0.007), duration of PBC of more than 1 year (*p* = 0.018), and incomplete release of the contracture (*p* = 0.002). The presence of keloids, hypertrophic scars, ulcers and the occurrence of complications at the contracture site were not associated with recurrence of PBC.

**Conclusion:**

Recurrence of PBC of the elbow and shoulder joint is a common problem. The risk factors should be kept in mind during management of PBC to reduce the recurrence rate.

**Electronic supplementary material:**

The online version of this article (doi:10.1186/s12893-015-0089-y) contains supplementary material, which is available to authorized users.

## Background

Post-burn contractures (PBC) are a distressing problem in both the developed and developing worlds [1–3]. They usually occur following inadequate primary burn injury management [4]. Children are the most affected by PBC, and the elbow and shoulder joints are the most involved regions [1, 5, 6]. Management of PBC of the elbow and shoulder joints is challenging, primarily because they have a tendency to recur [7–9].

Just as the inappropriate management of burns leads to the occurrence of PBC, inadequate management of PBC leads to their recurrence [1, 9, 10]. Recurrence has been reported in developed and developing countries following both surgical and non-surgical management [7–9]. Shoulder joint PBC have been reported to be more challenging to manage due to the wide range of motion of the joint. Management of these contractures has been reported to have complications which predispose to recurrences [11]. Elbow joint PBC are more likely to recur the longer the contractures have been present [3, 11], however, any joint involved by PBC, if inadequately managed, will have recurrence of the contracture.

There is a dearth of data available on the frequency of recurrence of PBC of the elbow and shoulder joint, especially in low-income countries such as Uganda. This study was carried out to determine the frequency of recurrence of PBC of the shoulder and elbow joint and the associated risk-factors among patients managed at our centre.

## Methods

### Study design

This was a prospective study involving all patients who had PBC of the elbow and/or shoulder joint who were managed at Comprehensive Rehabilitation Services Uganda (CoRSU) hospital from 2012 to 2014.

### Study setting

The study was conducted at the Plastic Surgery department of CoRSU Hospital, a specialised centre offering plastic and reconstructive surgery services in Uganda. It has a capacity of 84 beds and also serves as a training centre for the Mbarara University plastic surgery programme. CoRSU provides services to patients from the neighbouring towns and also the neighbouring countries like South Sudan, Kenya, Tanzania and D.R. Congo.

### Study population

The study population included patients of all age groups and sexes who had a PBC of the elbow and/or shoulder joint who were consecutively managed and reassessed at CoRSU hospital during the study period.

### Inclusion criteria

All patients with a PBC of the shoulder joint and/or elbow joint operated on at CoRSU hospital.

### Exclusion criteria

All patients who had severe disease co-morbidities, including malignancy (Marjolin’s ulcer) involving the affected limb, or with infection of the upper affected limb, were excluded from the study. All patients who were not residents of Uganda and were not to be followed up at CoRSU hospital were also excluded.

### Study materials

Data was collected using a coded questionnaire. A clear plastic goniometer with a 360 degree head and three scales calibrated to the International Standard of Measurements was used to measure the active range of motion of the affected joints.

### Study procedure

After obtaining informed consent, the participant’s socio-demographics, history of burn injury, and history of contracture were obtained. Examination of the affected joints for type of PBC, presence of ulcers, hypertrophic scars and/or keloids was done.

The active range of motion at each involved joint was measured using a manual goniometer with a standardized technique. The severity of contractures was recorded in degrees. Multiple planes of motion, i.e. flexion, extension, abduction, and adduction were measured at the involved shoulder joint(s) and recorded before surgery. Extension and flexion planes of motion were recorded before surgery for the elbow joint(s). The extension plane of motion was recorded as negative showing the extension deficit of the involved elbow joint.

After surgery the active range of motion of the operated joint(s) and the type of operative management, STSG, FTSG, Flap or a combination, were recorded. Any post-operative complications were recorded. The patients were seen by a physiotherapist and/or occupational therapist for splinting and mobilisation exercises. The patient was then discharged and given a date for follow-up.

After 1 month post-operation the patient’s use of splints, joint mobilisation regimen and supervision of physiotherapy were asked and recorded. The active range of motion of the operated joint(s) was measured using a goniometer. The presence of any complications was sought and recorded.

After 3 months post-operation the use of splints, joint mobilisation regimen and supervision of physiotherapy were again asked and recorded. The range of motion of the affected joint(s) was measured again. The presence of any complications at the operated joints was sought and recorded.

The information was entered in the database using Epidata Version 3.1. The range, consistency and validity checks were built in to minimize errors.

Elbow joint PBC were classified depending on the loss of joint extension as follows;

- Negligible, when there is less than 10 degrees of extension loss;
- Mild, when 11–49 degrees of extension loss
- Moderate, when there is 50–89 degrees of extension loss
- Severe when greater than 90 degrees of extension loss exists [3].

Shoulder joint PBC were classified depending on deficit in abduction as follows;

- Mild type; the arm abduction is above 150 degrees,
- Moderate type; limitation of abduction was from 120–150 degrees and the
- Severe type; limitation of abduction to less than 120 degrees [12].

The active range of motion of the joint measured after three months post operation was subtracted from the active range of motion obtained immediately post operation to get the degree of recurrence of post burn contracture.

### Ethical consideration

Clearance was obtained from the Faculty Research Ethics Committee of Mbarara University of Science and Technology (FREC-MUST) prior to commencement of the study and the patients or care givers gave informed consent to partake in the study.

### Statistical analysis

Data was exported to STATA version 12.1; stratification and univariate analysis was done. Poisson regression methods were used to estimate the overall incidence, risk and respective confidence intervals. A *p* value of < 0.05 was considered statistically significant.

## Results

During the study period 72 patients met the inclusion criteria, 4 patients were excluded because they were not to be followed up at CoRSU. 10 patients were lost to follow-up. 58 patients were analysed.

There were 36 females and 22 males, with a female-to-male ratio of 1.6:1. The age-range was 0.75–45 years, with a median age of 5 years. The average age at time of the initial burn injury was 3.4 years and the commonest cause of the initial burn injury was hot liquids (57 %). Flame burns affected 43 % of the participants [Table 1].

**Table 1:** Participants Characteristics

Characteristic	Participants’ distribution	
	Number	Percentage
Age categories in years		
≤5 years	29	51.79
>5 years	27	48.21
Gender		
Female	36	62.07
Male	22	37.93
Age categories at initial burn injury		
<1 year	11	18.97
1–5 years	36	62.07
>5 years	11	18.97
Cause of initial burn injury		
Flame	25	43.10
Hot Liquid	33	56.90
Treatment after initial burn injury		
No	2	3.45
Yes	56	96.55
Contracture duration in years		
≤1 year	28	48.28
>1 year	30	51.72
Number of large joints involved by PBCs		
Single	19	32.76
Multiple	39	67.24
Any previous contracture treatment		
No	52	89.66
Yes	6	10.34
Recurrence of elbow PBCs at initial presentation	50	96.15
No	2	3.85
Yes		
Recurrence of shoulder contracture at initial presentation.	54	93.10
No	4	6.90
Yes		

97 % of the participants had been treated in a hospital for the initial burn injury. 67 % of the participants had multiple-joint involvements, with the average number of joints involved by PBC being two. 10 % had prior treatment for post-burn contracture having already had recurrence, and of these 4 patients had recurrence at the shoulder joint and two at the elbow joint. More than half of the participants had had a contracture duration of over a year [Table 1]. The range of participant follow-up was 3 months to 2 years.

### Elbow joint

All the PBC were flexion contractures, with a right to left ratio of 1.2:1. Hypertrophic scars affected 15 % right and 26 % of left elbow joints. Keloids affected 11 % right and 13 % of the left elbow joints. No ulcers at the contracture site were observed. Additional file 1 shows a case of elbow post burn contracture and its managment with a local flap.

50 % of the elbow joint PBC were managed by skin grafting, and 50 % by local flaps, the commonest being Z-plasty. Complete release of the PBC was achieved in 57 % of the right elbow and 63 % of the left elbow joints [Table 2].

**Table 2:** Operative management employed and completeness of release

Patient treatment	PBC Shoulder		PBC Elbow	
	Right	Left	Right	Left
	*N* =14 (%)	*N* = 9 (%)	*N* = 28 (%)	*N* = 24 (%)
Operative management				
SSG	1 (7.14)	2 (22.22)	7 (25.00)	5 (20.83)
FTSG	0	0	7 (25.00)	4 (16.67)
Z-plasty	4 (28.57)	1 (11.11)	6 (21.43)	4 (16.67)
Y-V fap	3 (21.43)	2 (22.22)	2 (7.14)	2 (8.33)
Jumping man flap	5 (35.71)	1 (11.11)	2 (7.14)	4 (16.67)
Transposition flap	1 (7.14)	3 (33.33)	4 (14.29)	4 (16.67)
Bilobed flap	0	0	0	1 (4.17)
Completeness of Release of Pbc	*N* = 14	*N* = 9	*N* = 28	*N* = 24
No	9 (64.29)	6 (66.67)	12 (42.86)	9 (37.50)
Yes	5 (35.71)	3 (33.33)	16 (57.14)	15 (62.50)

### Shoulder joint

The right shoulder joint was more involved than the left shoulder joint, with a right to left ratio of 1.6:1. In the right shoulder joint the anterior fold (Type 1) was the most affected by the contracture where as in the left shoulder joint both the anterior and posterior folds were equally involved. Hypertrophic scars were present in over 50 % of both joints affected by PBC (57 % right side and 56 % left side). Keloids affected only 22 % of left shoulder joints and 7 % of the right shoulder joint.

The commonest operative technique employed was local flaps. Only 6 patients were managed by split skin grafting and none by full thickness grafts. Complete release of the contracture at the shoulder joint was achieved in 56 % of the right shoulder joint PBC and 33 % of the left joint contractures [Table 2].

The complications observed included wound dehiscence (14 %), wound infection (14 %), skin graft loss (7 %) and flap tip necrosis (7 %).

Patients managed by flaps had shorter hospital stays, approximately 6 days in total; compared to those managed by skin grafting, approximately 10 days. The difference in hospital stay was statistically significant (*p* = 0.009) [Table 3]. Among the patients managed by flaps, physiotherapy was started within 12 days of surgery compared to those managed by skin grafting where physiotherapy was started within 18 days. This difference was however, not statistically significant [Table 3].

**Table 3:** Length of Hospital Stay and Time prior to start of physiotherapy

	Mean (standard deviation) days				
	Overall	Among graft	Among flap	Mean difference	*p*-value
Hospital stay	7.49(5.70)	10.35(8.22)	5.97(2.92)	4.38	0.009
Time prior to Start of physiotherapy	14.18(10.68)	18.18(14.99)	12.06(6.89)	6.11	0.055

### Incidence of recurrence of PBC

Out of 58 participants, 30 patients (52 %) had a recurrence of the PBC. The frequency of recurrence of PBCs of the shoulder joint was 67 %, the elbow joint 27 %, and 100 % among participants who had a combination of elbow and shoulder joint PBC.

Risk factors were: flame as the cause of the initial burn (*p* = 0.007), duration of post burn contracture of more than one year (*p* = 0.018), and incomplete release of post burn contracture (*p* = 0.002).

## Discussion

In this study 30 participants (52 %) with PBC had a recurrence after operative management. The shoulder joint had a high frequency of recurrence at 67 %. Despite the frequency, the recurrent PBC of the shoulder joint were mostly of mild-to-moderate severity [Table 4]. The elbow joint had a lower frequency of recurrence (27 %) than the shoulder joint. Although the initial PBC of the elbow joint were mostly mild-to-severe, the recurrent contractures were mostly of a negligible-to-mild severity [Table 4]. This shows a general improvement in the active range of motion after operative management. Patients with a combination of elbow and shoulder joint PBC had 100 % recurrence of contractures.

**Table 4:** Severity of recurrence of post burn contractures

	Shoulder joint	
	Right (%)	Left (%)
Flexion		
Mild	12 (80.00)	5 (62.50)
Moderate	3 (20.00)	3 (37.50)
Severe	0	0
Extension		
Mild	15 (100)	8 (100)
Moderate	0	0
Severe	0	0
Abduction		
Mild	3 (20.00)	2 (25.00)
Moderate	6 (40.00)	3 (37.50)
Severe	6 (40.00)	3 (37.50)
Adduction		
Mild	15 (100)	8 (100)
Moderate	0	0
Severe	0	0
	Elbow Joint	
Extension		
Negligible	15 (55.56)	18 (75.00)
Mild	7 (25.93)	4 (16.67)
Moderate	3 (11.11)	2 (8.33)
Severe	2 (7.41)	0

Recurrence of PBC of the shoulder has been reported mostly following management of the contracture with skin grafting. The uneven axillary area presents a challenge for graft application as well as an increased occurrence of graft loss, flap loss and split-skin graft contraction after healing. This is the purported reason for the high recurrence of axillary contractures [11, 13, 14].

In our study the majority of patients with axillary PBC were managed by local flaps, however, we still observed post burn contracture recurrence. This finding was also reported by other authors [7]. In our study, the PBC of the axilla tended to be severe [Table 5] and residual contractures persisted after surgery [Table 2]. This was compounded by the limited appropriate use of splints; only 38 % of participants used splints, which may have led to recurrence of these contractures. The splints may have been inappropriately used because they were fashioned from Plaster of Paris. Our patients found these splints cumbersome and abandoned them while playing; this finding has been reported by other authors [14].

**Table 5:** Post Burn Contracture description before and after Operative management

Range of motion	Contracture descriptions			
	Shoulder joint			
	Right		Left	
	Before Treatment %	After Treatment %	Before Treatment %	After Treatment %
Flexion				
Mild	6 (42.86)	11 (78.57)	1 (11.11)	5 (55.56)
Moderate	7 (50.00)	3 (21.43)	5 (55.55)	4 (44.44)
Severe	1 (7.14)	0	3 (33.33)	0
Extension				
Mild	12 (85.71)	14 (100)	5 (55.56)	8 (88.88)
Moderate	1 (7.14)	0	3 (33.33)	1 (11.11)
Severe	1 (7.14)	0	1 (11.11)	0
Abduction				
Mild	1 (7.14)	4 (28.57)	0	3 (33.33)
Moderate	0	7 (50.00)	0	1 (11.11)
Severe	13 (92.86)	3 (21.43)	9 (100)	5 (55.55)
Adduction				
Mild	11 (78.57)	13 (92.86)	5 (55.56)	9 (100)
Moderate	2 (14.29)	1 (7.14)	4 (44.44)	0
Severe	1 (7.14)	0	0	0
	Elbow joint			
Extension				
Negligible	2 (7.14)	17 (60.71)	0	14 (58.33)
Mild	10 (35.71)	6 (21.43)	9 (37.50)	7 (29.17)
Moderate	10 (35.71)	3 (10.71)	11 (45.83)	3 (12.50)
Severe	6 (21.43)	2 (7.14)	4 (16.67)	0

The severity of the recurrent elbow joint PBC in our study was either negligible (<10 degrees extension deficit) or mild (between 11–49 degrees of extension loss) [Table 4]. This resulted in very little functional limitation, despite post burn contracture recurrence.

The highest incidence of recurrence was observed among patients who had a combination of both elbow and shoulder joint PBC. In our study, 100 % of participants experienced recurrence. All patients who presented to our centre with both elbow and shoulder joint PBC had the two contractures managed in a single operation. The practice of operating on both the elbow and the shoulder joints when a patient presents with a combination of PBC affecting these joints may have led to the high recurrence in this group. In our study, operating on both joints made post-operative management more demanding, especially the splinting of the joints in the anti-contracture positions and full mobilisation of these joints during physiotherapy. These challenges were especially salient in children. The practice of operating on as many joints as possible has been supported by other authors [11]. This practice is said to reduce costs for the patients and their families and the duration of anaesthetic exposure. However, other authors, such as Hudson et al. [15], recommend the release of the proximal joint followed by the distal joint, with each joint being released completely and rehabilitated to reduce recurrence of PBC.

The other reason for the high incidence of recurrence among the patients with both elbow and shoulder joint involvement may be due to the compensatory motions that patients with axillary joint PBC develop while performing activities of daily living. Plamieri et al. [17] showed that patients with post burn axillary contractures develop compensatory motions which include increased elbow flexion (to aid raising the hand over the head) and reduced internal rotation of the shoulder (to augment shoulder joint flexion). These compensatory motions, the authors believe, subject the patients to an increased risk of recurrence not only at the shoulder joint but also at the elbow joint.

We do believe the best practice may be to release each joint separately and completely, followed by adequate splinting and physiotherapy for the shoulder joint then the distal elbow joint. This would ensure adequate mobilisation of each operated joint and also prevent the compensatory motions that tend to occur in patients who have both axillary and elbow joint PBC.

Factors associated with recurrence of PBC in our study included: flame as the cause of the initial burn injury (*p* = 0.007), duration of contracture of more than one year (*p* = 0.018) and failure to achieve complete release of the contracture (*p* = 0.002). Patients with PBC following flame burn were twice as likely to have recurrence compared to those who had had scalds. Flame burns are usually deep dermal or mixed thickness compared to scalds [16]. Because of this flame, burns may lead to more severe PBC that, if managed inappropriately, could lead to recurrence of the contracture.

Duration of post burn contracture of more than one year was associated with recurrence of PBC. This is contrary to other studies which advocate for the release of the contracture after one year [10, 11]. The waiting period of one year is supposedly to allow the post-burn scar to mature, which is said to reduce the recurrence rate. PBC of a longer duration lead to severe shortening of the musculotendinous units, the joint capsule and the neurovascular structures, which may predispose the patients to recurrence of contractures and our findings are in agreement. However, Schwarz et al. [11] reported that in children a complete range of motion can be expected in spite of the duration of contracture, as long as the operation is done before the child is 12 years old.

Failure to achieve complete release of the contracture was associated with a high risk of recurrence (*p* = 0.002). The residual contractures were observed in cases of severe contracture of long standing duration, in which there was severe shortening of the underlying structures. This made it challenging to safely release the contracture in a single stage. The residual contractures may also have led to compensatory motions especially at the shoulder joint increasing the tendency of recurrence [17].

Physiotherapy has been reported to reduce the incidence of recurrence by ensuring active range of motion of the affected joint [3, 18, 19]. However, in our study, some of the patients who reported having had physiotherapy had a recurrence of PBC. This may be because following wound healing the physiotherapy was started in-hospital and supervised by a therapist. However, upon reaching home, there was no longer any supervision by a qualified therapist. This is shown in our study, where patients’ attendants were the supervisors of the physiotherapy in 57 % of cases, and a qualified therapist in only 10 %. We believe the physiotherapy at home may not have been performed adequately on a daily basis due in part to inadequate supervision and poor pain management.

Factors which were not associated with an increased risk of recurrence of PBC at a statistically significant level included: Age at initial burn injury (*p* = 0.671), presence of keloids (*p* = 0.802), hypertrophic scars (*p* = 0.1644), ulcers at the contracture site (*p* = 0.142), and presence of complications (*p* = 0.103) (including infection, graft loss, wound dehiscence and flap tip necrosis).

### Limitation of the study

The duration of follow-up of may not have been enough to determine the frequency of recurrence in all the participants.

The immediate post-operative range of motion could have been limited by post-operative pain and tissue oedema.

The measurement of active range of motion of a joint by goniometry does not translate into the equivalent limitation of recurrent PBC on the activities of daily living. Some patients may be functioning well even with some limitation in the active range of motion as measured by goniometry.

Burning agents in the study were limited to flame and hot liquid.

Materials that have been shown to reduce recurrence of PBC like Acellular dermal matrix (ADM) and Integra were not used due to their prohibitive cost.

## Conclusion

Recurrence of PBC of the elbow and shoulder joint is common. Patients who were initially burnt by flame, have contractures for more than one year, have involvement of both the shoulder and elbow joint, and/or have incomplete release of contracture during surgery, are at an increased risk for recurrence.

Surgeons managing PBC of the elbow and/or shoulder joint should take into consideration the associated risk factors and modify operative and post-operative management accordingly. Appropriate splinting, physiotherapy, supervision of the physiotherapy regimen and splint usage should be done to reduce the recurrence rate of PBC.

### Recommendation

PBC should be released within the first 12 months if at all possible.

Shoulder joint PBC should be released with flap coverage followed by appropriate use of splints and physiotherapy.

The presence of keloids, hypertrophic scars or ulcers at the contracture site should not deter or delay management of PBC.

## Additional file

Additional file 1:
**4.5 year old child with an elbow post burn contracture secondary to scald injury.** He was managed with jumping man flap with good results.
