# Supplementary material for: Recurrence of post burn contractures of the elbow and shoulder joints: experience from a ugandan hospital
Source: BMC Surg. 2015 Sep 9;15:103. doi: 10.1186/s12893-015-0089-y (PMC4564967; doi:10.1186/s12893-015-0089-y)

Case .1.

4.5 year old male child with an elbow joint Post burn contracture . cause of the primary injury was a scald.

Pre-operative markings of a patient with post burn contracture of the elbow and Carpometacarpal joint of the thumb.


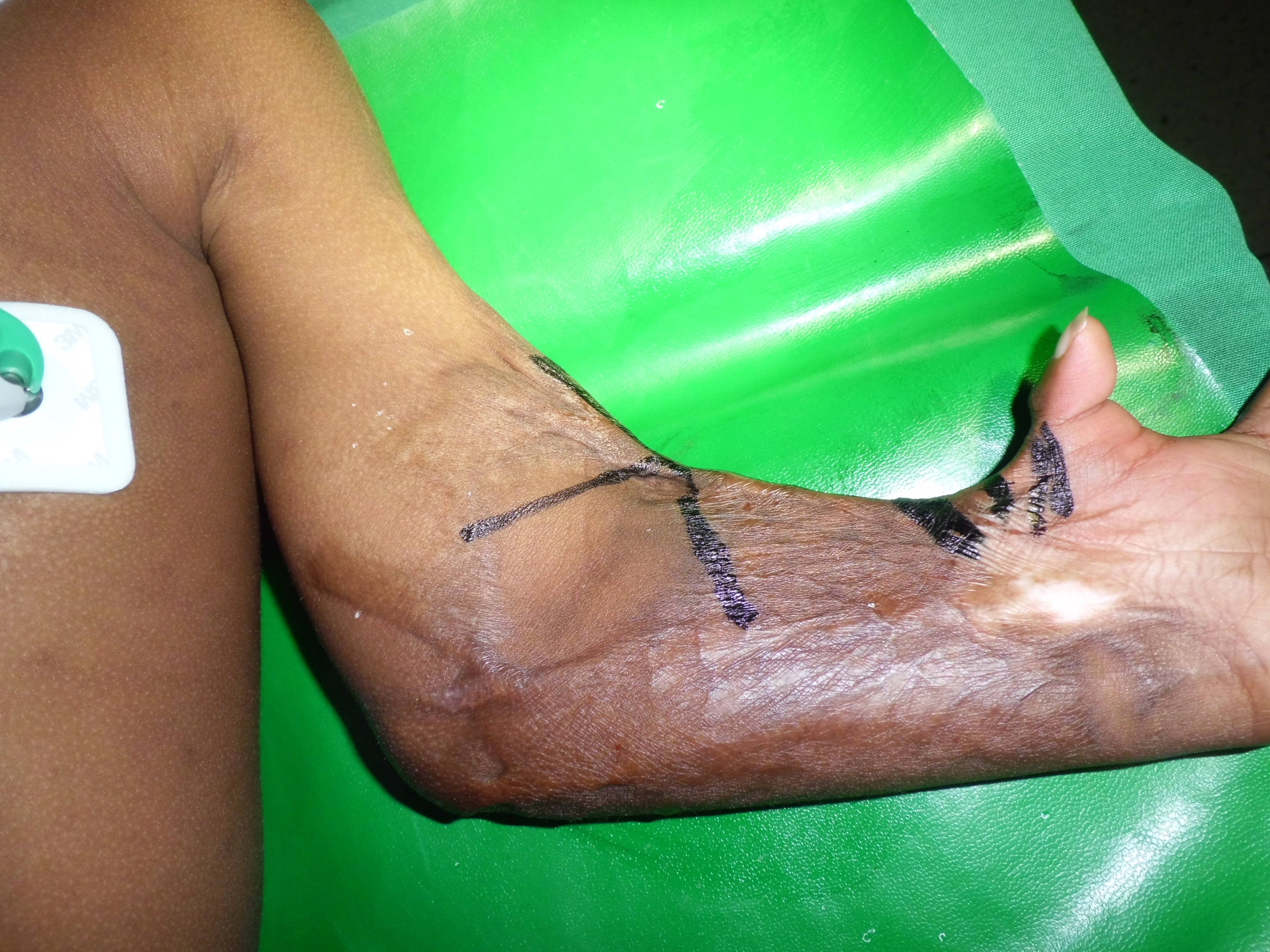


Jumping man flap marked out for the elbow joint and the extension contracture of the carpometacarpal joint of the thumb.


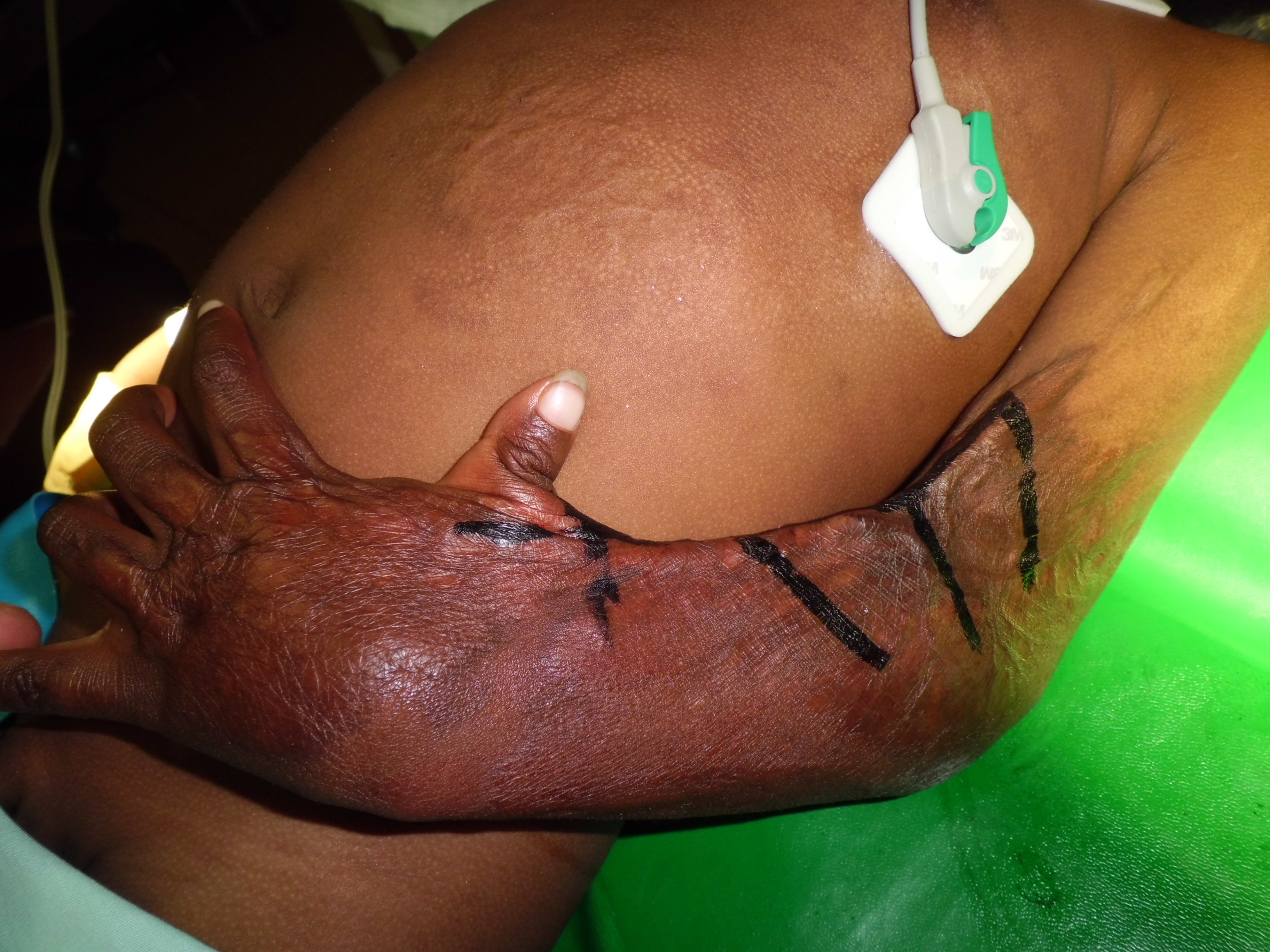


Post operative view in the clinic.


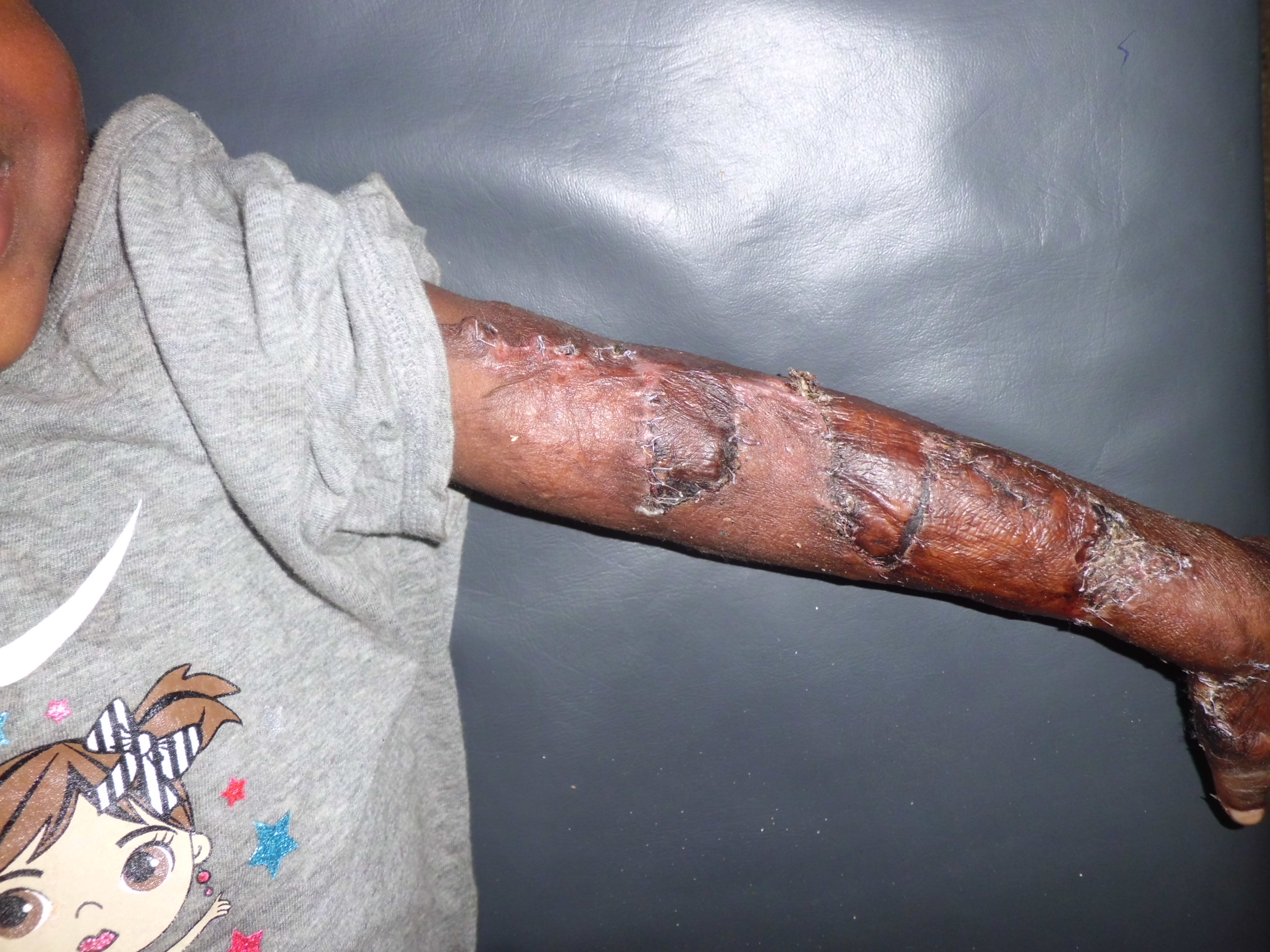

Supplement: Additional file 1: — 4.5 year old child with an elbow post burn contracture secondary to scald injury. He was managed with jumping man flap with good results. [file 12893_2015_89_MOESM1_ESM.doc]
